# A randomized double-blind placebo-controlled trial of an inhibitor of plasminogen activator inhibitor-1 (TM5614) in mild to moderate COVID-19

**DOI:** 10.1038/s41598-023-50445-1

**Published:** 2024-01-02

**Authors:** Toyohiro Hirai, Koichiro Asano, Isao Ito, Yasunari Miyazaki, Hisatoshi Sugiura, Mehmet Agirbasli, Seiichi Kobayashi, Makoto Kobayashi, Daishi Shimada, Ichiro Natsume, Tsutomu Kawasaki, Takehiko Ohba, Sakurako Tajiri, Fumio Sakamaki, Masamichi Mineshita, Takahisa Takihara, Kiyoshi Sekiya, Keisuke Tomii, Hiromi Tomioka, Hideo Kita, Yasuo Nishizaka, Motonari Fukui, Toshio Miyata, Hideo Harigae

**Affiliations:** 1 Department of Respiratory Medicine, Kyoto University Graduate School of Medicine, 54 Shogoin-Kawaharacho, Sakyo, Kyoto 606-8507 Japan; 2 Division of Pulmonary Medicine, Department of Medicine, Tokai University School of Medicine, Kanagawa, Japan; 3 Department of Respiratory Medicine, Tokyo Medical and Dental University, Tokyo, Japan; 4 Department of Respiratory Medicine, Tohoku University Graduate School of Medicine, Sendai, Japan; 5 Department of Cardiology, Istanbul Medeniyet University Hospital TR, Istanbul, Turkey; 6 Department of Respiratory Medicine, Japanese Red Cross Ishinomaki Hospital, Ishinomaki, Japan; 7 Department of Respiratory Medicine, Osaki Citizen Hospital, Osaki, Japan; 8 Department of Infectious Disease Medicine, Tohoku Medical and Pharmaceutical University, Sendai, Japan; 9 Department of Respiratory Internal Medicine, Yokosuka Kyosai Hospital, Yokosuka, Japan; 10 Department of Respiratory Medicine, Yokohama City Minato Red Cross Hospital, Yokohama, Japan; 11 Department of Respiratory Medicine, Ome Municipal General Hospital, Ome, Japan; 12 Department of Respiratory Medicine, Tokai University Oiso Hospital, Oiso, Japan; 13 Department of Respiratory Medicine, Tokai University Hachioji Hospital, Hachioji, Japan; 14 Department of Respiratory Medicine, St. Marianna University School of Medicine, Kawasaki, Japan; 15 Department of Respiratory Medicine, Ebina General Hospital, Ebina, Japan; 16 Department of Allergy and Respiratory Medicine, National Organization Sagamihara National Hospital, Sagamihara, Japan; 17 Department of Respiratory Medicine, Kobe City Medical Center General Hospital, Kobe, Japan; 18 Department of Respiratory Medicine, Kobe City Medical Center West Hospital, Kobe, Japan; 19 Department of Respiratory Medicine, Takatsuki Red Cross Hospital, Takatsuki, Japan; 20 Department of Respiratory Medicine, Osaka Red Cross Hospital, Osaka, Japan; 21 Department of Respiratory Medicine, Medical Research Institute Kitano Hospital, Osaka, Japan; 22 Department of Molecular Medicine and Therapy, Tohoku University Graduate School of Medicine, 2-1 Seiryo-Machi, Aoba-Ku, Sendai, Miyagi 980-8575 Japan; 23 Department of Hematology, Tohoku University Graduate School of Medicine, Sendai, Japan

**Keywords:** Viral infection, Randomized controlled trials

## Abstract

An inhibitor of plasminogen activator inhibitor (PAI)-1, TM5614, inhibited thrombosis, inflammation, and fibrosis in several experimental mouse models. To evaluate the efficacy and safety of TM5614 in human COVID-19 pneumonia, phase IIa and IIb trials were conducted. In an open-label, single-arm trial, 26 Japanese COVID-19 patients with mild to moderate pneumonia were treated with 120–180 mg of TM5614 daily, and all were discharged without any notable side effects. Then, a randomized, double-blind, placebo-controlled trial was conducted in Japanese COVID-19 patients with mild to moderate pneumonia. The number of study participants was set to be 50 in each arm. Even after extension of the enrollment period, the number of study participants did not reach the initially intended sample size, and 75 patients were enrolled in the study. The total oxygenation scale from Day 1 to Day 14 as the primary endpoint was 1.5 in the TM5614 group vs 4.0 in the placebo group (p = 0.22), and the number of days of oxygen administration required as the secondary endpoint was 2.0 days in the TM5614 group vs 3.5 days in the placebo group (p = 0.34). Further studies will be necessary to verify the efficacy of PAI-1 inhibition for the treatment of COVID-19 pneumonia.

**Clinical trial registration**: Two studies were conducted: a prospective, multicenter, open-label phase II study at https://jrct.niph.go.jp (jRCT2021200018) (First registration date 18/08/2020) and a prospective, multicenter, randomized, double-blind, placebo-controlled, phase II study at https://jrct.niph.go.jp (jRCT2021210006) (First registration date 28/05/2021).

## Introduction

The novel coronavirus (SARS-CoV-2) infectious disease (COVID-19) has been both a medical and a social issue. Most infected people have mild symptoms, but patients with high-risk factors, such as elderly persons and patients with underlying diseases (e.g., diabetes mellitus and kidney disease), can present with severe pneumonia and acute respiratory distress syndrome (ARDS)^1^. Measures have been taken to allow mild cases to be treated at home, but the existence of some cases that present with mild disease at onset and rapidly become severe is a distressing problem. Development of a low-molecular-weight therapeutic drug that is safe, conveniently administered orally for outpatients, and prevents aggravation of pneumonia would not only prolong the lives of patients, but also contribute to a decreased burden on the healthcare system.

In patients with severe pneumonia caused by COVID-19, lesions such as inflammation and fibrosis progress rapidly, and, in particular, characteristic findings of hypercoagulability are also observed^2^. A highly characteristic finding of COVID-19 pneumonia is fibrin microthrombi in the lung^3^. It has been reported that there is a significant correlation between hypercoagulability and mortality in patients infected with SARS-CoV-2, characterized by high blood t-PA/PAI-1 and D-dimer levels^4^. Lung injury caused by COVID-19 is primarily explained by dramatic alveolar endothelial damage leading to ARDS with microvascular thrombosis^5^. Therefore, drugs that have anti-thrombotic and fibrinolytic activities are expected to be effective.

TM5614 is a small molecule inhibitor of plasminogen activator inhibitor (PAI)-1 that we developed. Based on the crystal structure of human PAI-1, TM5614 was explored by in silico drug discovery and selected from more than 1,400 novel derivatives^6–8^. Unlike other anti-thrombotic agents, the anti-thrombotic and fibrinolytic benefits of PAI-1 inhibitors are obtained without adversely affecting bleeding in experimental animals including monkeys; they do not change activated partial thromboplastin time and prothrombin time or platelet activity and do not prolong bleeding time^9^. In a phase II clinical trial of TM5614, 180 mg/day was administered for 48 weeks to 33 patients with chronic myelogenous leukemia, and there were no serious adverse events caused by the investigational drug^10^. TM5614 is thus a drug with high safety in humans.

Previous preclinical studies demonstrated that, in addition to anti-thrombotic and fibrinolytic effects, PAI-1 inhibitors provide clinical benefits for lung injuries, *i.e.,* inhibiting bleomycin- and TGF-β-induced lung fibrosis^6,11^, improving lung inflammation^6^ and emphysema in the Klotho aging and *N*^*ϖ*^-nitro-L-arginine methyl ester-induced lung models^12–14^, and protecting alveolar epithelial cells^15^. Recently, Kang et al*.* demonstrated that the inhibition of IL-6 signaling by tocilizumab treatment decreased PAI-1 production and alleviated clinical manifestations of severe COVID-19 patients^16^. Unlike tocilizumab (antibody drug), TM5614 is a low-molecular-weight drug that is orally available and inhibits the activity of PAI-1.

A series of clinical studies were therefore conducted in Japan to evaluate the efficacy and safety of PAI-1 inhibitors for COVID-19 pneumonia, *i.e*., an open-label, early phase II clinical trial and a randomized, double-blind, placebo-controlled, late phase II clinical trial in COVID-19 patients with mild to moderate pneumonia. This paper for the first time reports the results of clinical trials of PAI-1 inhibition for the treatment of COVID-19 pneumonia.

## Methods

### Open-label study

#### Study design and patients

An early phase II safety and efficacy clinical study was conducted at seven hospitals in Japan between October 2020 and March 2021. It was registered with the Japan Registry of Clinical Trials (https://jrct.niph.go.jp; registration No. jRCT2021200018; first registration date 18/08/2020). Eligible patients were hospitalized adults (age ≥ 20 years) with COVID-19 with mild to moderate pneumonia, as confirmed by a positive RT-PCR or a rapid antigen test of respiratory specimens. Patients also had to meet the inclusion criteria listed below. The study protocol was approved by the Institutional Review Board (IRB) of Tohoku University (No. 203004), as well as by the IRBs of all participating institutions. After eligibility was assessed, patients were given a detailed explanation and signed an informed consent form.

#### Inclusion criteria

Inclusion criteria were as follows:

1. Hospitalized patients 20 years of age or older who signed the informed consent form,
2. Patients who were positive for SARS-CoV-2 by methods based on gene amplification, including a PCR test or antigen test,
3. Patients with findings suggestive of COVID-19 pneumonia on computed tomography (CT),
4. Patients with oxygen saturation < 95% at rest and on room air,
5. Patients requiring supplemental oxygen therapy < 5 L/min,
6. Patients not connected to a ventilator,
7. Patients with AST or ALT ≤ 2.5 times the upper limit of the institutional standard,
8. Patients with a total bilirubin value ≤ 2.5 times the upper limit of the institutional standard,
9. Patients with creatinine clearance > 30 mL/min.

Patients were excluded if they were receiving home-oxygenation therapy or dialysis for chronic renal disease, had active malignant disease or inactive malignant disease for less than 5 years, Child–Pugh class B or C liver cirrhosis, a bleeding tendency, if they were pregnant or breastfeeding, or if they could not discontinue or had to use the contraindicated drugs listed in Supplementary Table 1.

#### Procedures

TM5614 was administered orally at a starting dose of 120 mg once daily, which was increased to 180 mg from Day 8 as tolerated. The planned duration of treatment was 14 days. Standard treatments were given according to the treatment protocol for COVID-19 of the Ministry of Health, Labour and Welfare of Japan. Within the scope of the study, no hospitalization or any extra examinations were performed other than as part of standard care.

#### Outcomes

The primary endpoint was usage of mechanical ventilation after treatment with TM5614.

The secondary endpoints included:

1. Survival on Day 28,
2. Length of hospital stay after the start of the study,
3. Number of days supplemental oxygen was required,
4. P/F ratio (PaO_2_/FiO_2_),
5. Changes in lung lesions on chest computed tomography (CT).

The degree of lung damage on chest CT was determined by two methods, as follows.

The chest CT severity score was evaluated semiquantitatively to visually assess the severity of COVID-19 pneumonia. In 6 lung segments, percent areas of involvement such as ground-glass opacities (GGOs), crazy-paving, and consolidation were scored as follows: 0%—score 0; < 25%—score 1; 25 to < 50%—score 2; 50 to < 75%—score 3; and ≥ 75%—score 4. The sum of all 6 lung segment scores was obtained.

Quantitative analysis using image analysis software (AI-based quantitative CT image analysis software (AIQCT)) was performed to quantify the following 10 images: normal lung, bronchial shadow, vascular shadow, reticular shadow, GGO, honeycomb lung, consolidation, hyperlucent lung, granular shadow, and others^17^.

#### Statistical analysis

Data for continuous parameters are reported as mean ± standard deviation or median with interquartile range values, depending on the distribution of the variable. Categorical variables are presented as percentages (%). For continuous variables, patterns of distribution were analyzed with a visual inspection of histograms and with the Shapiro–Wilk test. Comparisons between groups were done with Student’s *t-*test or the Mann–Whitney U test for variables with parametric or nonparametric distribution, respectively. For categorical variables, the chi-square test or Fisher’s exact test was used to compare groups. A p-value < 0.05 was considered significant. All statistical analyses were done with SPSS 25.0 statistical software (IBM Inc., Armonk, NY, USA).

### Randomized, double-blind, placebo-controlled study

A late phase II, safety and efficacy clinical study of TM5614 was then conducted after approval by the Institutional Review Board (IRB) of Tohoku University (No. 213002), as well as by the IRBs of all participating institutions.

#### Study design and patients

This randomized, double-blind, placebo-controlled, late phase II clinical trial was conducted at 20 hospitals in Japan. It was registered with the Japan Registry of Clinical Trials (https://jrct.niph.go.jp; registration No. jRCT2021210006; First registration date 28/05/2021). The number of study subjects was set to be 50 in each arm based on the calculations shown in the statistical analysis section, with the initially planned study period to go from June 24, 2021 to February 28, 2022. Due to a shortage of enrolled subjects with the end of the local epidemic wave of COVID-19, the study period was extended to September 30, 2022. Eligible patients and inclusion criteria were the same as for the “Open-label study” above. After eligibility was assessed, written, informed consent was obtained from all participants.

#### Procedures

Following enrollment, eligible patients were randomly assigned to two treatment arms in a 1:1 ratio to take orally either 120 mg or 180 mg of TM5614 or equivalent numbers of placebo tablets once daily. Placebo tablets were indistinguishable in appearance and packaging from TM5614 tablets. Study participants, care providers, and members of the outcome assessment committee were all blinded. An independent, unmasked person generated the treatment allocation sequence. Blinded, randomly serialized packages composed of 6 treatment drug sets with labelling numbers were prepared, and a package was distributed to each hospital. In each hospital, blinded medical doctors enrolled participants, and a blinded pharmacist assigned each participant to a set of drugs in numerical order. The planned duration of treatment was 14 days. Standard treatments were given in the same manner as for the “Open-label study” above.

#### Outcomes

The primary endpoint was the sum of the six-category oxygenation scale recorded daily from Days 1 to 14. The oxygenation scale observed for the longest time of the day was recorded every day, and the total score for 14 days was used as the index. The six-category oxygenation scale was defined as follows: 0, no oxygen required; 1, nasal or mask oxygenation ≤ 2 L/min; 2, nasal or mask oxygenation > 2 L/min, < 5 L/min; 3, nasal or mask oxygenation ≥ 5 L/min or oxygenation with a reservoir mask; 4, high-flow nasal cannula oxygenation or non-invasive positive-pressure ventilation required; and 5, invasive mechanical ventilation with extracorporeal membrane oxygenation (ECMO) required, or death.

The secondary endpoints included the following, in addition to the 5 items in the “Open-label study”:

1. Percentage of cases showing oxygenation scale 4 or higher,
2. Changes in plasma D-dimer levels at enrollment and on Day 7,
3. Percentage of cases with clinical thrombosis (deep vein thrombosis, cerebral infarction, myocardial infarction, or others) developing after enrolment,
4. Percentage of cases requiring anticoagulant therapy including heparin,
5. Total oxygenation scale corrected by the Comprehensive Clinical Characterisation Collaboration (4C) Deterioration model^18^,
6. Efficacy evaluation using NIAID’s 7-point ordinal scale.

The investigational drug was considered effective if the patient was discharged or improved by at least 2 points on the following NIAID-defined 7-point ordinal scale from Day 1 to Day 7. The ordinal scale was: 1, death; 2, hospitalized with intubated ventilation or ECMO; 3, hospitalized with noninvasive positive pressure ventilation or nasal high-flow cannula; 4, hospitalized with low-flow oxygen; 5, hospitalized with no need for oxygen, but receiving continuous medical care; 6, hospitalized with no need for oxygen or continuous medical care (except as specified in this study); and 7, discharged from the hospital.

#### Safety

Adverse events were coded using MedDRA/J Ver. 20.0 and classified according to system organ class (SOC) and preferred term (PT).

#### Statistical analysis

Statistical analysis was performed using SAS Release 9.4 TS Level 1 M5 (64-bit) or later. The TM5614 and placebo groups were compared as for the “Open-label study” above. When the amount of change before and after treatment was assessed, analysis of covariance was performed using the values before treatment as covariates.

#### Plasma activities of tPA and PAI-1

For measurement of tPA activity, plasma samples were collected with sodium citrate (pH 4.3) at a final concentration of 50 mM and promptly frozen below − 80 °C for storage after centrifugation. Plasma tPA activity was measured using a ZYMUPHEN™ tPA activity ELISA kit (Hyphen Biomed, Paris, France). For measurement of PAI-1 activity, plasma samples were collected using vacuum blood collection tubes containing 3.2% or 3.13% Na citrate and promptly frozen at or below − 80 °C for storage after centrifugation. Plasma PAI-1 activity was measured using a Human PAI-1 Activity ELISA Kit (Molecular Innovations, Novi, MI, USA). The percentage of patients with either a decrease in PAI-1 activity or an increase in tPA activity from Day 1 to Day 7 was tested for significant differences between the TM5614 and placebo groups using Pearson’s chi-square test.

### Ethical approval statement

These studies were conducted in accordance with the Declaration of Helsinki and Good Clinical Practice and were approved by the Ethics Committee of Tohoku University (No. 203004 and 213002) and by the ethics committees of all participating institutions for the Japanese studies.

### Patient consent information

All patients provided written, informed consent before enrollment.

## Results

### Open-label study

Twenty-nine patients were enrolled in the study, including one who dropped out after screening and two who withdrew consent. Twenty-six patients were included in the full analysis set (FAS) (Supplementary Table 2). All 26 patients started taking 120 mg once daily, and 18 patients received an increased dose (from 120 to 180 mg) on Day 8.

#### Efficacy

In the 26 patients, there was no worsening of oxygenation requiring ventilator management (the primary endpoint). As for the secondary endpoints, 25 patients were alive, but no survival information was available at 28 days for one patient. The median length of hospitalization after the initiation of the investigational drug was 15 days (mean ± standard deviation, 15.2 ± 3.5 days) (n = 22). The median number of days of oxygenation required after the initiation of the investigational drug was 7 days (8.4 ± 6.1 days) (n = 26).

A total of 18 patients underwent chest CT both at enrollment and on Day 14. The total CT score of the semiquantitative scoring system for lung lesions was 9.1 ± 3.0 at the beginning of treatment and 5.8 ± 3.6 on Day 14, and it showed a significant improvement in lung lesions by Day 14 (significant reduction: -3.3 (95% confidence interval: -5.14 to -1.26), p = 0.0018) (Supplementary Fig. 1). The results for other secondary endpoints are presented in the supplementary text.

#### Safety

Of the 26 patients, 42 adverse events were observed in 19 patients (73.1%). Of these, the causal relationship to the study drug was undeniable for 17 adverse events in 10 patients (38.5%), but no serious adverse events were observed. Details of the adverse events are shown in Supplementary Table 3.

#### Concomitant medications

Concomitant medications included favipiravir in 5 patients, remdesivir in 8 patients, steroids in 22 patients, and antiplatelet agents in 5 patients, but no serious adverse events were observed. Unfractionated heparin is used in Japan as a treatment for COVID-19 patients. There was no experience with the combined use of unfractionated heparin and TM5614 before, and combined use was prohibited in this clinical trial protocol. Unfractionated heparin was used in 2 patients, and they were discontinued from the trial.

### Randomized, double-blind, placebo-controlled study

**Figure 1:**
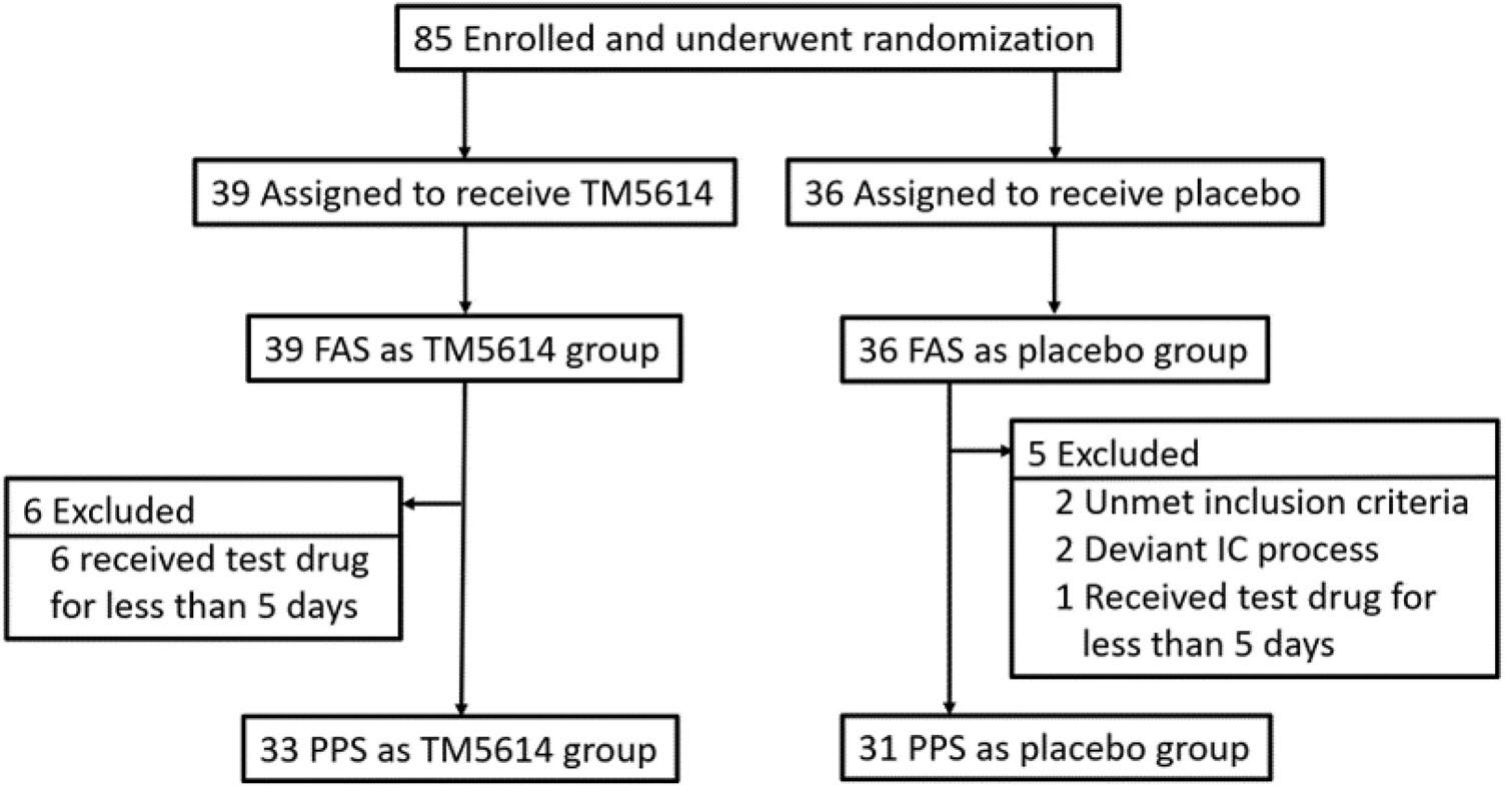
Enrollment and randomization (randomized, double-blind, placebo-controlled study). *FAS* full analysis set, *IC* informed consent, *PPS* per-protocol set.

Seventy-five patients with mild to moderate pneumonia were randomized to the TM5614 group (N = 39) and the placebo group (N = 36) and included in the FAS (Fig. 1, Table 1). All of these patients started taking 120 mg once daily, and 57 patients received an increased dose (from 120 to 180 mg) from Day 8. Sixty-four patients were included in the per-protocol set (PPS).

**Table 1:** Demographic data and baseline characteristics (randomized, double-blind, placebo-controlled study).

		TM5614		Placebo		p-value
		Number	%	Number	%	
Sex	Male	27	(69.2%)	27	(75.0%)	0.62^b^
	Female	12	(30.8%)	9	(25.0%)	
Age when obtaining consent (y)	20–39	1	(2.6%)	6	(16.7%)	–
	40–59	25	(64.1%)	20	(55.6%)	–
	60–79	10	(25.6%)	8	(22.2%)	–
	≥ 80	3	(7.7%)	2	(5.6%)	–
	Mean ± SD	58.1 ± 12.5	–	53.3 ± 14.8	–	0.13^a^
	Median (Min, Max)	56.0 (30, 84)	–	55.5 (24,80)	–	–
BMI (kg/m^2^)	< 18.5	0	(0.0%)	1	(2.8%)	–
	≥ 18.5 but < 25	20	(51.3%)	11	(30.6%)	–
	≥ 25	19	(48.7%)	24	(66.7%)	–
	Mean ± SD	25.3 ± 4.3	–	27.1 ± 5.2	–	0.10^a^
	Median (Min, Max)	24.3 (18.6, 38.8)	–	25.9 (17.2, 40.1)	–	–
Medical history	None	20	(51.3%)	19	(52.8%)	1.00^b^
	Yes	19	(48.7%)	17	(47.2%)	
Comorbidity	None	6	(15.4%)	6	(16.7%)	1.00^b^
	Yes	33	(84.6%)	30	(83.3%)	
Smoking history	None	17	(43.6%)	15	(41.7%)	1.0^b^
	Yes	22	56.4%)	21	(58.3%)	
SpO_2_ (on oxygen) (%)	Mean ± SD	94.7 ± 1.9	–	94.5 ± 1.7	–	0.66^a^
	Median (Min, Max)	95 (89, 99)	–	95 (89, 98)	–	–
Symptom: Dyspnea (mMRC)	Grade 0	4	(10.3%)	6	(16.7%)	0.47^b^
	Grade 1	10	(25.6%)	14	(38.9%)	
	Grade 2	10	(25.6%)	7	(19.4%)	
	Grade 3	9	(23.1%)	7	(19.4%)	
	Grade 4	6	(15.4%)	2	(5.6%)	
Symptom: Cough	None	5	(12.8%)	10	(27.8%)	0.15^b^
	Yes	34	(87.2%)	26	(72.2%)	
Symptom: Sputum	None	20	(51.3%)	22	(61.1%)	0.49^b^
	Yes	19	(48.7%)	14	(38.9%)	
Oxygen scale	(0/1/2)	(13/18/8)		(13/19/4)		0.53^c^
The scores of the 4C Deterioration Model	Mean ± SD	427.7 ± 117.8		395.4 ± 117.3		0.24^a^
Chest X-ray findings	No abnormality	2	(5.13%)	1	(2.78%)	1.00^b^
	Abnormality	37	(94.9%)	35	(97.2%)	
Chest CT findings	No abnormality	0	(0.0%)	0	(0.0%)	1.00^b^
	Abnormality	39	(100.0%)	36	(100.0%)	
CT Visual scores	Mean ± SD	8.5 ± 3.5		6.9 ± 3.1		0.051^d^
CT Normal lung area (%)	Mean ± SD	64.2 ± 17.9		66.5 ± 18.7		0.54^a^

^a^Unpaired *t*-test.

^b^Fisher’s exact test.

^c^Chi-square test.

^d^Mann-Whitney U test.

#### Efficacy

Results for the primary and secondary endpoints are shown in Table 2. The total oxygenation scale from Day 1 to Day 14 in the FAS as the primary endpoint was 1.5 in the TM5614 group and 4 in the placebo group (p = 0.22). The number of days oxygen was required in the FAS as the secondary endpoint was 2 days in the TM5614 group and 3.5 days in the placebo group (p = 0.34).

**Table 2:** Primary and secondary endpoints (randomized, double-blind, placebo-controlled study).

Endpoint	TM5614 (n = 39)	Placebo (n = 36)	Risk ratio (95% CI)	p-value
Primary endpoint				
Total oxygenation scale from Day 1 to Day 14, FAS	1.5 [0, 5]; n = 30	4.0 [2, 6]; n = 31	–	0.22
Secondary endpoint				
Survival on day 28 (%)	29/29 (100)	31/31 (100)	N/A	1.00
Length of hospital stay after the start of the study (days)	10.7 ± 6.2	11.8 ± 5.9	–	0.50
Number of days oxygen required, FAS (days)	2 [1, 3]; n = 39	3.5 [2, 5]; n = 36	–	0.34
Change of P/F ratio (PaO_2_/FiO_2_) from enrollment to Day 14	291.53 ± 63.45; n = 3	541.70; n = 1	–	ND
Changes in lung lesions on chest computed tomography (CT) images from enrollment to Day 14				
Change in the visual score	− 3.5 ± 4.4; n = 8	-1.2 ± 2.7; n = 12	–	0.27
Change in the normal lung area (%)	25.4 ± 21.8; n = 6	15.0 ± 22.2; n = 12	–	0.13
Cases showing 4 or higher on the oxygenation scale (%)	4/39 (10.3)	0/36 (0)	N/A	0.12
Changes in plasma D-dimer levels at enrollment and on Day 7, adjusted mean ± SE (μg/mL increase)	1.47 ± 1.08; n = 31	1.90 ± 1.07; n = 32	–	0.78
Cases with clinical thrombosis (deep vein thrombosis, cerebral infarction, myocardial infarction, or others) that presented after enrollment (%)	1/39 (2.6)	1/36 (2.8)	0.92 (0.06–14.22)	1.0
Cases requiring anticoagulant therapy including heparin (%)	6/39 (15.4)	1/36 (2.8)	5.54 (0.70–43.80)	0.11
Total oxygenation scale corrected by 4C Deterioration model, adjusted mean ± SE	4.0 ± 0.9; n = 30	5.3 ± 0.9; n = 31	–	0.29
Efficacy evaluation using NIAID’s 7-point ordinal scale, effective (%)	4/31 (12.9)	7/33 (21.2)	0.61 (0.20–1.88)	0.51

Values are indicated as medians [95% CI] or means ± SD.

**Figure 2:**
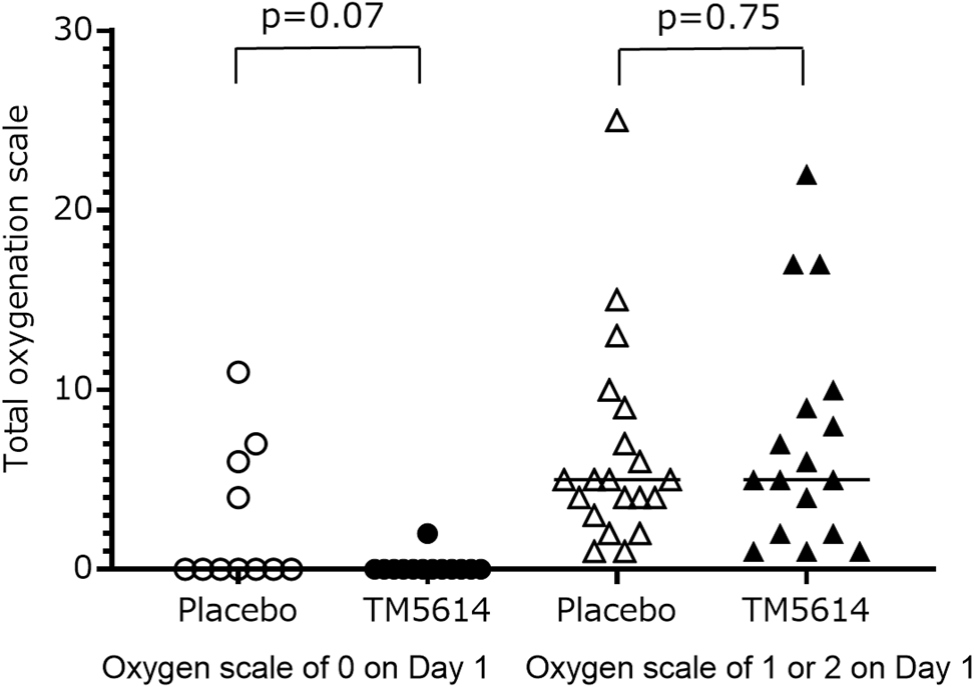
Effect of TM5614 or placebo evaluated by the total oxygen score from Day 1 to Day 14 (randomized, double-blind, placebo-controlled study). In the hospitalized patients with early pneumonia who were not receiving oxygen, the total oxygenation scale from Day 1 to Day 14 is marginally lower in the TM5614 group than in the placebo group (p = 0.07). This is not the case in patients receiving oxygen (p = 0.75).

In the subgroup analysis, the total oxygenation scale was lower in the TM5614 group (n = 14) than in the placebo group (n = 16) in the subgroup with the 4C deterioration score ≤ median score (0.5 ± 0.9 vs 3.0 ± 3.4, p = 0.03), but it was not different between the two groups (n = 16 and 15, respectively) in the subgroup with the 4C deterioration score > median score (7.3 ± 6.5 vs 7.3 ± 6.4, p = 0.98). Similarly, the scale was lower in the TM5614 group than in the placebo group in the subgroup not requiring oxygen at enrollment (p = 0.07) (Fig. 2). However, this was not the case in the subgroup with moderate pneumonia requiring oxygen (p = 0.75).

**Figure 3:**
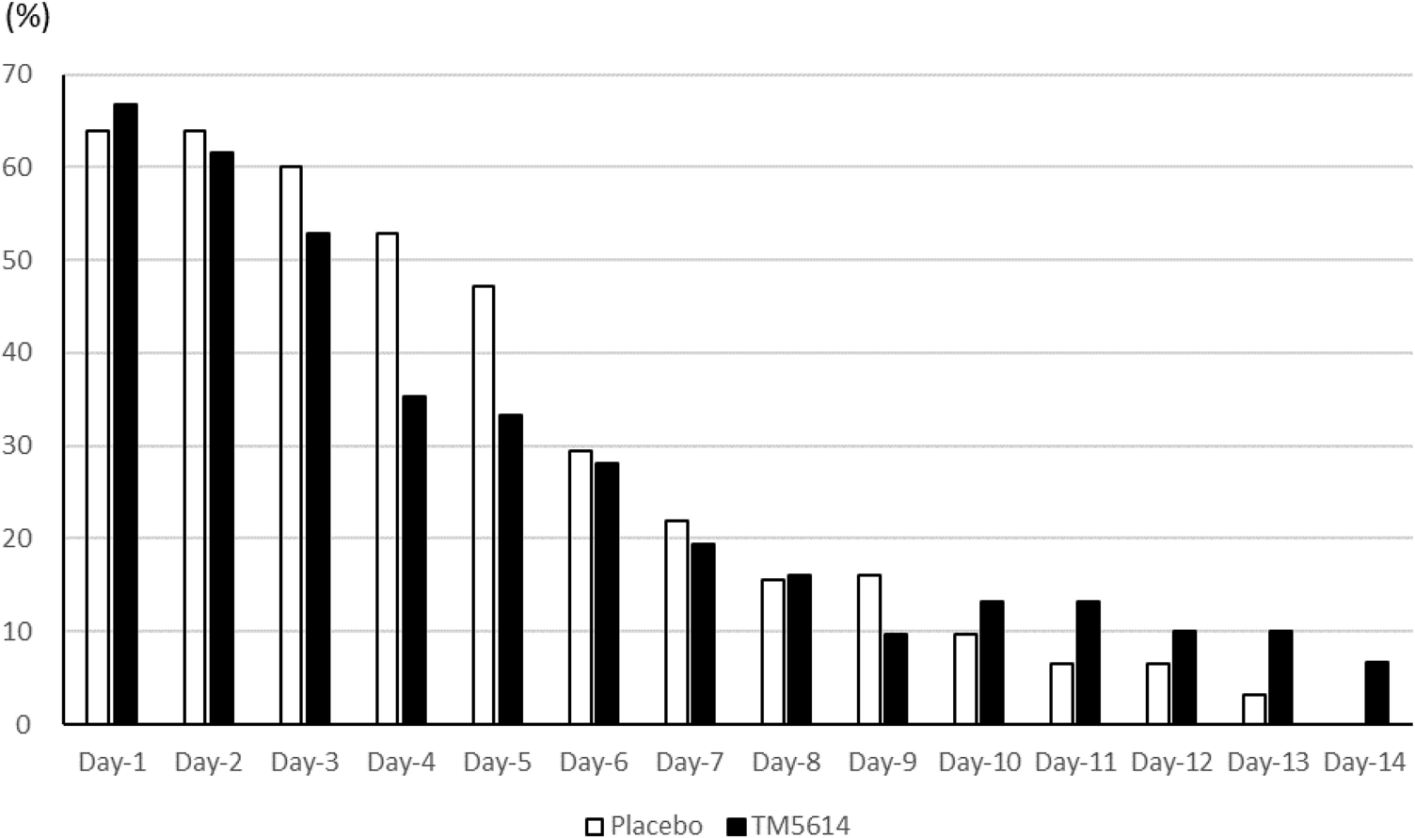
Proportion of patients requiring oxygen therapy (randomized, double*-*blind, placebo*-*controlled study). The proportions of patients with the oxygenation scale of 1 or higher on Days 3, 4, and 5 in the FAS are 52.8%, 35.3%, and 33.3%, respectively, in the TM5614 group, and 60.0%, 52.9%, and 47.1%, respectively, in the placebo group (p = 0.38, 0.14, and 0.25, respectively).

Proportions of patients with the oxygenation scale of 1 or higher on Days 3, 4, and 5 were 52.8%, 35.3%, and 33.3%, respectively, in the TM5614 group, and 60.0%, 52.9%, and 47.1%, respectively, in the placebo group (p = 0.38, 0.14, and 0.25, respectively) (Fig. 3).

A total of 20 patients underwent chest CT both at enrollment and on Day 14. The semiquantitative visual score for lung lesions showed improvement in the TM5614 group (median value of changes in the total score = − 3.5 ± 4.4; n = 8), compared with those in the placebo group (median value of changes in the total score = − 1.2 ± 2.7; n = 12). On quantitative analysis of the chest CT images, two cases, one obtained with contrast medium and one with a difference in the field of view (FOV) greater than 10% between the first and second scans, were excluded. The normal lung area increased from 56.7% before treatment to 82.1% after treatment in the TM5614 group (n = 6, 25.4 percentage point improvement), whereas the improvement in the placebo group was 15.0 percentage points (n = 12, from 60.5% to 75.5%) (Supplementary Fig. 2). Although there was no significant difference between the two groups (p = 0.13), TM5614 appeared to improve lung injury on chest CT. The GGO areas, which are characteristic lesions of COVID-19, decreased more in the TM5614 group, by 24.1 percentage points (from 31.0% before treatment to 6.9% after treatment), than in the placebo group, by 13.9 percentage points (from 24.3% to 10.4%), though with no significant difference. The results for other secondary endpoints are presented in the supplementary text.

#### Biomarkers

**Figure 4:**
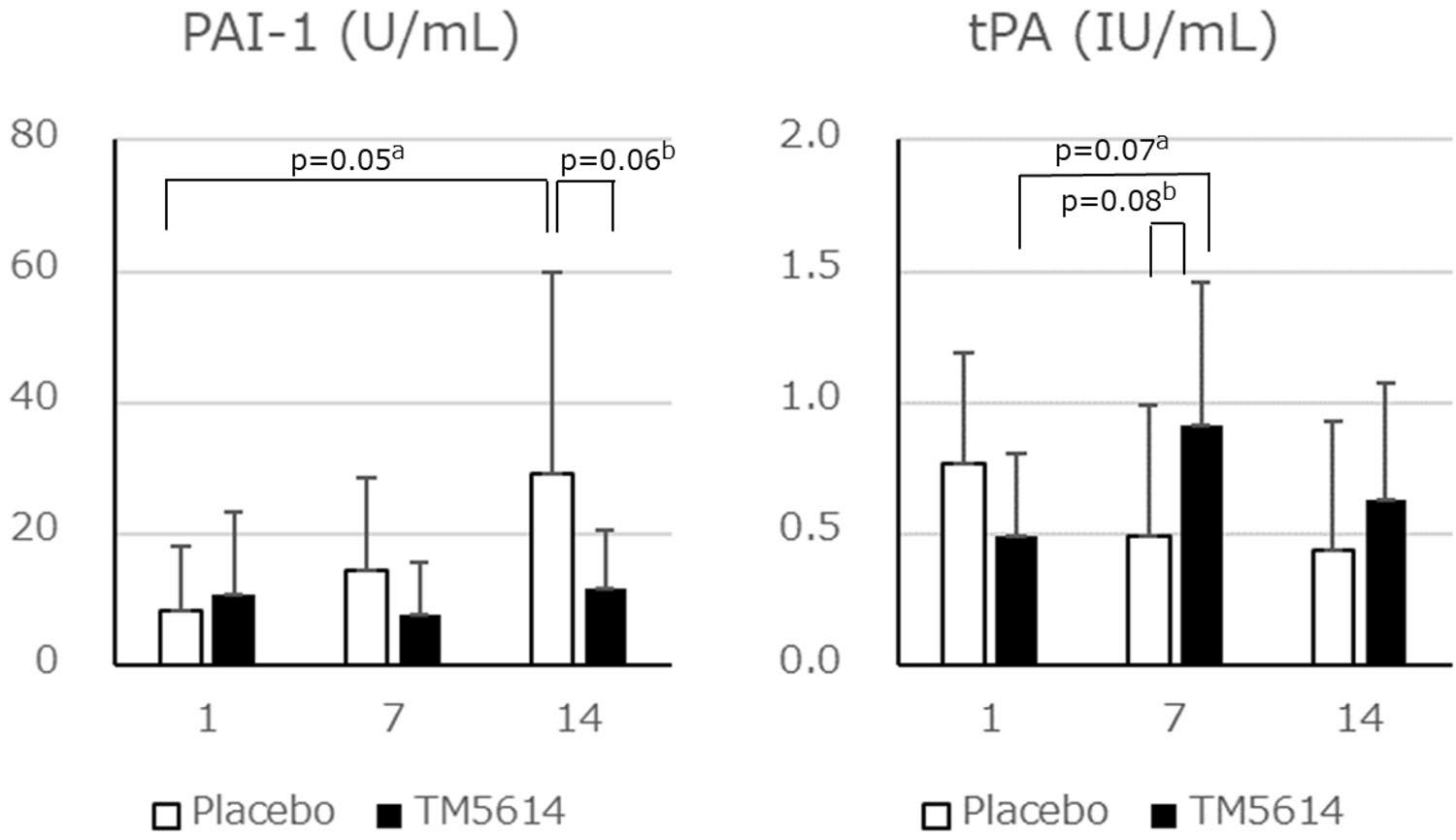
Plasma activities of PAI-1 and tPA (randomized, double-blind, placebo-controlled study). The plasma activities of tPA and PAI-1 in patients were measured on Days 1, 7, and 14 (n = 9 in the TM5614 group and n = 6 in the placebo group). a: p-value by one-sided paired *t*-test and b: p-value by one-sided Student’s *t*-test.

The plasma activities of PAI-1 and tPA were measured in 15 patients (9 in the TM5614 group and 6 in the placebo group) (Fig. 4). PAI-1 activity increased marginally from Day 1 to Day 14 in the placebo group (p = 0.05), whereas it did not change in the TM5614 group, suggesting that TM5614 suppressed activation of PAI-1 induced by SARS-CoV-2. In contrast, tPA activity increased marginally from Day 1 to Day 7 in the TM5614 group (p = 0.06), whereas it did not change in the placebo group, suggesting that TM5614 could have a pro-fibrinolytic effect even in the pro-coagulation environment of COVID-19.

#### Safety

Of the adverse events observed in 22 of 39 patients (38 cases) in the TM5614 group (56.4%) and in 23 of 36 patients (42 cases) in the placebo group (63.9%), side effects were reported in 7 patients (11 cases in which the relationship to the investigational drug could not be ruled out) in the TM5614 group (17.9%) and 10 patients (13 cases in which a relationship could not be ruled out and 1 case of nasal bleeding that was related to the investigational drug) in the placebo group (27.8%). Cases showing ≥ 4 on the oxygenation scale were 4/39 (10.3%) in the TM5614 group vs 0/36 (0%) in the placebo group (p = 0.12), which are considered to be unlikely related to the TM5614. Severe or serious adverse event related to TM5614 were not observed, confirming the safety of TM5614 in patients with pneumonia. Details of the adverse events are shown in Supplementary Table 3.

#### Concomitant medications

Favipiravir in 3 patients (0 in TM5614, 3 in placebo), remdesivir in 45 (24 in TM5614, 21 in placebo) patients, steroids in 66 (34 in TM5614, 32 in placebo) patients, and antiplatelet agents in 10 (6 in TM5614, 4 in placebo) patients were used, but no serious adverse events were observed. Overall, 61.5% (24/39) of the TM5614 group and 69.4% (25/36) of the placebo group had been vaccinated for COVID-19 before enrollment, and no difference in efficacy was observed between patients with and without SARS-CoV-2 vaccination.

## Discussion

Various novel treatments for COVID-19 have been established and investigated throughout the last two years, including antivirals, immunomodulators, monoclonal antibodies to SARS-CoV-2, and antithrombotic agents. However, studies are needed to improve our understanding of the final common pathways that lead to the clinical sequelae in severe COVID-19, and there is still a need to identify novel therapies to mitigate the severity of lung injury associated with SARS-CoV-2 infection.

In patients with pneumonia caused by SARS-CoV-2, lesions such as inflammation and fibrosis progress rapidly, and, in particular, characteristic findings of hypercoagulability are observed^2^. Kang et al*.* demonstrated that the inhibition of IL-6 signaling by tocilizumab treatment decreased PAI-1 production and alleviated clinical manifestations in severe COVID-19 patients^16^. Several studies reported a close link between PAI-1 and lung fibrosis^6,11^. Studies in experimental models of lung injury reported that elevated levels of PAI-1 initiate lung injury^19–21^. Therefore, drugs against PAI-1 that act on anti-thrombotic and fibrinolytic systems could be expected to be effective for the treatment of COVID-19 pneumonia.

We developed an inhibitor of plasminogen activator inhibitor (PAI)-1, TM5614. The crystallographic structure analysis has shown that the binding site of TM5614 within the PAI-1 molecule is the binding site of vitronectin^22^. Since the binding of vitronectin to PAI-1 is essential for the stabilization of the PAI-1 molecule, TM5614 may shorten the functional half-life of PAI-1 and may affect its clearance rate by inhibiting the binding of vitronectin. We demonstrated that fine particulate matter (PM2.5) increased circulating levels of PAI-1 and pro-inflammatory cytokine interleukin-6 in bronchoalveolar lavage fluid of an experimental model of air pollution, which was inhibited by TM5614 treatment^23^. In a bleomycin-induced lung fibrosis animal model, PAI-1 inhibitors such as siRNA to silence PAI-1 resulted in significant downregulation of PAI-1 expression and decreased collagen deposition in the lung^24^. Several other animal models of lung injury showed that PAI-1 and fibrinolytic system components regulate alveolar type 2 cell and fibroblast apoptosis^19–21,25^. Pharmacological inhibition of PAI-1 protects against vascular and lung damage related to PAI-1^19–21,25^. In preclinical studies, in addition to anti-thrombotic and fibrinolytic benefits, PAI-1 inhibitors inhibit bleomycin- and TGF-β-induced lung fibrosis^6,11^, prevent lung inflammation^6^, and improve lung emphysema in the Klotho aging and $N^{{\overline{\omega }}}$-nitro-L-arginine methyl ester-induced lung models^12–14^. Thus, TM5614 was expected to prevent worsening of pneumonia caused by SARS-CoV-2.

In the present report, 26 Japanese patients hospitalized with moderate lung injury were first treated with 120–180 mg of TM5614 daily, and all were discharged without any notable side effects. A similar open-label study was also conducted with the same protocol with four COVID-19 patients with moderate pneumonia in Turkey from December 2020 to January 2021 (approved by the Turkiye Republic Ministry of Health, Turkish Medicines and Medical Devices Agency-Regulatory and Supervisory Authority, TITCK protocol number 20-AKD-83). All four patients recovered from COVID-19 uneventfully and were discharged home.

A placebo-controlled trial was then conducted in 75 hospitalized COVID-19 Japanese patients with mild to moderate pneumonia. After the start of vaccination, the COVID-19 disease course and phenotype changed significantly. Similarly, novel variants, effective therapies, and the global response have evolved in the course of the pandemic. After the four waves of the COVID-19 epidemic in Japan, the Delta and Omicron variants have reached global circulation. In June 2021, we started a multicenter, placebo-controlled study at 20 medical institutions involving hospitalized patients with moderate pneumonia in Japan and expected to enroll 100 patients in total. Initially, the major prevailing viral strain was the Delta variant, and by the end of September 2021, more than half of the target number of patients had been registered. However, after the Omicron strain became the mainstream strain, the number of patients with COVID-19 pneumonia, excluding unvaccinated cases, decreased, the number of patients eligible for this trial decreased sharply, and enrollment decreased significantly. Therefore, enrollment was cut-off in October 2022, and the total number of patients enrolled was 75.

In the randomized, placebo-controlled study, the primary endpoint was the total oxygenation scale from Day 1 to Day 14 (TM5614 1.5 vs placebo 4.0), and the secondary endpoint was the number of days of oxygen required (TM5614 2.0 vs placebo 3.5 days). Although the differences were not significant due to the early cut-off of enrollment, the usefulness of TM5614 was suggested in patients with early pneumonia who were not receiving oxygen, in contrast to patients who were receiving oxygen. The proportion of cases requiring oxygen therapy was also lower in the TM5614 group 3–5 days after hospitalization. CT findings evaluated by the normal lung area suggested improvement was more prominent in the TM5614 group, though not significantly. Regarding cases that received anticoagulation therapy, the initial 4C mortality scores of 5 of 6 cases in the TM5614 group were above the median value, whereas the initial 4C mortality score of the only case in the placebo group was also above the median value. This suggests that, in the milder half of cases with 4C mortality scores ≤ the median value, there was no difference between the two groups in anticoagulant usage. Taken together, TM5614 is expected to have clinical benefits, especially in patients with early pneumonia.

Moreover, the present results are in good agreement with our preclinical data showing that the therapeutic effect of TM5614 was higher when administered early in the bleomycin-induced lung fibrosis animal model. Moeller et al. reported that this model is associated with the inflammatory phase (≤ 7 days after the last bleomycin application), followed by the fibrosis phase (> 7 days after the last bleomycin application)^26^. TM5614 given in the inflammatory phase provides significant therapeutic benefits in this model (Hirai T and Sato A, unpublished observation). Measures have been taken to allow mild cases to be treated at home or stay in the hospital overnight, but the existence of some patients who are mild at the onset of the disease and rapidly become severe is an emerging problem. Since TM5614 is safe and conveniently administered orally, it may provide clinical benefits for outpatients with pneumonia in the inflammatory phase by preventing the aggravation of pneumonia.

Various novel treatments for COVID-19 have been established and investigated throughout the last few years, including vaccines, antivirals, immunomodulators, monoclonal antibodies to SARS-CoV-2, and antithrombotic agents. In the present study, several different concomitant medications were used. Antivirals, such as favipiravir and remdesivir, and steroids have completely different mechanisms of action from TM5614. Antivirals such as favipiravir and remdesivir, steroids, and antiplatelet agents were used concomitantly with TM5614, but no serious adverse events were observed. Low-molecular-weight heparin was used in four Turkish patients, and early start of menstrual bleeding in a female patient was reported. Thus, TM5614 could be a potential option for treatment in combination with the established drugs. Since SARS-CoV-2 has not been vanquished, and worldwide COVID-19 epidemic waves can still be expected in the future, there continues to be a persistent need for orally-available drugs to suppress development of severe disease.

The reported studies have several limitations. First, in the randomized study, the number of study subjects did not reach the target despite extension of the study period. This was mainly because cases of pneumonia appropriate for enrollment, *i.e.* without predetermined underlying diseases, decreased dramatically along with the prevalence shift to the Omicron variant of SARS-CoV-2. Second, there were medical advances against COVID-19 such as vaccination and antiviral drugs during the study period. In the phase IIb study, 65.3% (49/75) of the enrolled patients had received vaccination against COVID-19, and 60% (45/75) and 88% (66/75) were treated with remdesivir and steroids concomitantly with TM5614. Considering that vaccination, remdesivir, and steroids have been proven to be effective in preventing worsening of the disease, and that many of the mild to moderate disease cases could be self-healing in nature, such treatments would have masked the clinical effect of TM5614. Moreover, vaccination or the concomitant treatments could have potentially affected the therapeutic outcomes of test drugs. However, the double-blinding and randomization of the present study would have minimized their influence in the comparison between the TM5614 group and the placebo group.

In conclusion, initial observations of pharmacological inhibition of PAI-1 in moderate cases of COVID-19 pneumonia were reported. Given the deleterious effects of high PAI-1 levels in COVID-19 patients, it is encouraging that the novel therapeutic agent TM5614 that can potentially accelerate the fibrinolytic system could be a treatment option for COVID-19. TM5614 is a safe, orally administered drug that has different mechanisms of action from the established drugs. Future studies with a large number of patients are needed to further establish PAI-1 inhibition as a rationale to slow the accelerated lung injury in acute COVID-19.

### Supplementary Information

Supplementary Information.

## Data Availability

The data that support the findings of this study are available from the corresponding author, T.H., upon reasonable request. The study protocols of phase IIa and IIb trails are available on the trial websites https://jrct.niph.go.jp/en-latest-detail/jRCT2021200018 and https://jrct.niph.go.jp/en-latest-detail/jRCT2021210006, respectively.

## References

[1] Wu Z, McGoogan JM (2020). Characteristics of and important lessons from the coronavirus disease 2019 (COVID-19) outbreak in china: Summary of a report of 72314 cases from the Chinese Center for Disease Control and Prevention. JAMA.

[2] Tang N, Li D, Wang X, Sun Z (2020). Abnormal coagulation parameters are associated with poor prognosis in patients with novel coronavirus pneumonia. J. Thromb. Haemost..

[3] Ackermann M, Verleden SE, Kuehnel M, Haverich A, Welte T, Laenger F, Vanstapel A, Werlein C, Stark H, Tzankov A, Li WW, Li VW, Mentzer SJ, Jonigk D (2020). Pulmonary vascular endothelialitis, thrombosis, and angiogenesis in Covid-19. N. Engl. J. Med..

[4] Jin X, Duan Y, Bao T, Gu J, Chen Y, Li Y, Mao S, Chen Y, Xie W (2020). The values of coagulation function in COVID-19 patients. PLoS One.

[5] Ciceri F, Beretta L, Scandroglio AM, Colombo S, Landoni G, Ruggeri A, Peccatori J, D’Angelo A, De Cobelli F, Rovere-Querini P (2020). Microvascular COVID-19 lung vessels obstructive thromboinflammatory syndrome (MicroCLOTS): An atypical acute respiratory distress syndrome working hypothesis. Crit. Care Resusc..

[6] Izuhara Y, Takahashi S, Nangaku M, Takizawa S, Ishida H, Kurokawa K, van Ypersele de Strihou C, Hirayama N, Miyata T (2008). Inhibition of plasminogen activator inhibitor-1: Its mechanism and effectiveness on coagulation and fibrosis. Arterioscler. Thromb. Vasc. Biol..

[7] Yamaoka N, Kawano Y, Izuhara Y, Miyata T, Meguro K (2010). Structure-activity relationships of new 2-acylamino-3-thiophenecarboxylic acid dimers as plasminogen activator inhibitor-1 inhibitors. Chem. Pharm. Bull. (Tokyo).

[8] Yamaoka N, Kodama H, Izuhara Y, Miyata T, Meguro K (2011). Structure-activity relationships of new N-acylanthranilic acid derivatives as plasminogen activator inhibitor-1 inhibitors. Chem. Pharm. Bull. (Tokyo).

[9] Izuhara Y, Yamaoka N, Kodama H, Dan T, Takizawa S, Hirayama N, Meguro K, van Ypersele de Strihou C, Miyata T (2010). A novel inhibitor of plasminogen activator inhibitor-1 provides antithrombotic benefits devoid of bleeding effect in nonhuman primates. J. Cereb. Blood Flow Metab..

[10] Takahashi N, Kameoka Y, Onizuka M, Onishi Y, Takahashi F, Dan T, Miyata T, Ando K, Harigae H (2022). Deep molecular response in patients with chronic phase chronic myeloid leukemia treated with the plasminogen activator inhibitor-1 inhibitor TM5614 combined with a tyrosine kinase inhibitor. Cancer Med..

[11] Huang WT, Vayalil PK, Miyata T, Hagood J, Liu RM (2012). Therapeutic value of small molecule inhibitor to plasminogen activator inhibitor-1 for lung fibrosis. Am. J. Respir. Cell. Mol. Biol..

[12] Eren M, Boe AE, Murphy SB, Place AT, Nagpal V, Morales-Nebreda L, Urich D, Quaggin SE, Budinger GR, Mutlu GM, Miyata T, Vaughan DE (2014). PAI-1-regulated extracellular proteolysis governs senescence and survival in Klotho mice. Proc. Natl. Acad. Sci. U. S. A..

[13] Boe AE, Eren M, Murphy SB, Kamide CE, Ichimura A, Terry D, McAnally D, Smith LH, Miyata T, Vaughan DE (2013). Plasminogen activator inhibitor-1 antagonist TM5441 attenuates Nω-nitro-L-arginine methyl ester-induced hypertension and vascular senescence. Circulation.

[14] Boe AE, Eren M, Morales-Nebreda L, Murphy SB, Budinger GR, Mutlu GM, Miyata T, Vaughan DE (2015). Nitric oxide prevents alveolar senescence and emphysema in a mouse model. PLoS One.

[15] Rana T, Jiang C, Liu G, Miyata T, Antony V, Thannickal VJ, Liu RM (2020). PAI-1 Regulation of TGF-β1-induced alveolar type II cell senescence, SASP secretion, and SASP-mediated activation of alveolar macrophages. Am. J. Respir. Cell Mol. Biol..

[16] Kang S, Tanaka T, Inoue H, Ono C, Hashimoto S, Kioi Y, Matsumoto H, Matsuura H, Matsubara T, Shimizu K, Ogura H, Matsuura Y, Kishimoto T (2020). IL-6 trans-signaling induces plasminogen activator inhibitor-1 from vascular endothelial cells in cytokine release syndrome. Proc. Natl. Acad. Sci. U. S. A..

[17] Handa T, Tanizawa K, Oguma T, Uozumi R, Watanabe K, Tanabe N, Niwamoto T, Shima H, Mori R, Nobashi TW, Sakamoto R, Kubo T, Kurosaki A, Kishi K, Nakamoto Y, Hirai T (2022). Novel Artificial intelligence-based technology for chest computed tomography analysis of idiopathic lung fibrosis. Ann. Am. Thorac. Soc..

[18] Gupta RK, Harrison EM, Ho A (2021). Development and validation of the ISARIC 4C deterioration model for adults hospitalized with COVID-19: A prospective cohort study. Lancet Respir. Med..

[19] Wang J, Fang C, Wang S, Fang F, Chu X, Liu N, Lu C, Wang S, Li W (2020). Danggui Buxue Tang ameliorates bleomycin-induced lung fibrosis in rats through inhibiting transforming growth factor-β1/Smad3/ plasminogen activator inhibitor-1 signaling pathway. J. Tradit. Chin. Med..

[20] Jiang C, Liu G, Cai L, Deshane J, Antony V, Thannickal VJ, Liu RM (2021). Divergent regulation of alveolar type 2 cell and fibroblast apoptosis by plasminogen activator inhibitor 1 in lung fibrosis. Am. J. Pathol..

[21] Puthusseri B, Marudamuthu A, Tiwari N, Fu J, Idell S, Shetty S (2017). Regulation of p53-mediated changes in the uPA-fibrinolytic system and in lung injury by loss of surfactant protein C expression in alveolar epithelial cells. Am. J. Physiol. Lung Cell. Mol. Physiol..

[22] Sillen M, Miyata T, Vaughan DE, Strelkov SV, Declerck PJ (2021). Structural insight into the two-step mechanism of PAI-1 inhibition by small molecule TM5484. Int. J. Mol. Sci..

[23] Ghosh AK, Soberanes S, Lux E, Shang M, Aillon RP, Eren M, Budinger GRS, Miyata T, Vaughan DE (2021). Pharmacological inhibition of PAI-1 alleviates cardiopulmonary pathologies induced by exposure to air pollutants PM2.5. Environ. Pollut..

[24] Ding L, Zhu C, Yu F, Wu P, Chen G, Ullah A, Wang K, Sun M, Li J, Oupický D (2018). Pulmonary delivery of polyplexes for combined PAI-1 gene silencing and CXCR4 inhibition to treat lung fibrosis. Nanomedicine.

[25] Wigén J, Löfdahl A, Bjermer L, Elowsson-Rendin L, Westergren-Thorsson G (2020). Converging pathways in lung fibrosis and Covid-19—The fibrotic link to disease severity. Respir. Med. X.

[26] Moeller A, Ask K, Warburton D, Gauldie J, Kolb M (2008). The bleomycin animal model: A useful tool to investigate treatment options for idiopathic lung fibrosis?. Int. J. Biochem. Cell Biol..

