# Supplementary material for: A randomized double-blind placebo-controlled trial of an inhibitor of plasminogen activator inhibitor-1 (TM5614) in mild to moderate COVID-19
Source: Sci Rep. 2024 Jan 2;14:165. doi: 10.1038/s41598-023-50445-1 (PMC10761996; doi:10.1038/s41598-023-50445-1)
Supplement: Supplementary file 1 — Supplementary Information. [file 41598_2023_50445_MOESM1_ESM.docx]

**Supplement**

**Supplementary text**

**Results**

I. Open-label study

Efficacy

The P/F ratio as a secondary endpoint was not evaluated because the data available for analysis were insufficient.

The change in the quantitative analysis of CT images using image analysis software (difference between Day 1 and Day 14) was a 17.2±24.1% (n=18) increase in normal lung area (from 57.2±19.5% to 74.4±12.7%), indicating that the area of normal lung increased significantly.

**Figure legends**

**Supplementary Figure 1. Total CT score of the semiquantitative scoring system for lung lesions (open-label study in Japan)**

The chest CT severity score was evaluated semiquantitatively at enrollment and on Day 14 to visually assess the severity of COVID-19 pneumonia (n=18). In 6 lung segments, percent areas of involvement such as ground-glass opacities (GGOs), crazy-paving, and consolidation were scored as follows: 0% - score 0; < 25% - score 1; 25 to <50% - score 2; 50 to <75% - score 3; and ≥ 75% - score 4. The sum of all 6 lung segments scores was obtained.

**Supplementary Figure 2. Normal lung area (%) on quantitative analysis of chest CT (randomized, double-blind, placebo-controlled study)**

Normal lung areas (%) are compared between before treatment (enrolled) and Day 14 in the TM5614 group and the placebo group (paired *t*-test). Increases in normal lung areas are compared between the TM5614 group and the placebo group (ANCOVA).

**Supplementary Table 1. Contraindicated drugs**

| 1. Contraindicated in phase IIa | |
| --- | --- |
|  | Warfarin potassium |
|  | Dabigatran etexilate methanesulfonate |
|  | Rivaroxaban |
|  | Apixaban |
|  | Edoxaban tosilate hydrate |
|  | Heparin, low-molecular-weight heparin |
|  | Recombinant tissue plasminogen activator |
|  | Nafamostat mesilate |
|  | Camostat mesilate |
| 1. Contraindicated in phase IIb | |
|  | Drugs listed in 1. |
|  | Danaparoid sodium |
|  | Fondaparinux sodium |
|  | Baricitinib |
|  | Vaccines for SARS-CoV-2 |

Administration of these drugs was prohibited for 5 days after initiation of the study. If contraindicated drugs were given to subjects on Days 0-5, their participation in the trial was terminated. If these drugs were given after Day 6, administration of the test drug was stopped, but data acquisition continued.

**Supplementary Table 2. Demographic data and baseline characteristics (open-label study)**

|  |  | Number | Percentage |
| --- | --- | --- | --- |
| Sex | Male | 18 | (69.2%) |
|  | Female | 8 | (30.8%) |
| Age when obtaining consent (y) | 20-39 | 1 | (3.8%) |
|  | 40-59 | 13 | (50.0%) |
|  | 60-79 | 10 | (38.5%) |
|  | ≥80 | 2 | (7.7%) |
|  | Mean±SD | 60.2±14.0 | |
|  | Median (Min, Max) | 58.5 (36, 91) | |
| BMI | <18.5 | 1 | (3.8%) |
| (kg/m^2^) | ≥18.5 but <25 | 8 | (30.8%) |
|  | ≥25 | 17 | (65.4%) |
|  | Mean±SD | 27.1±7.2 | |
|  | Median (Min, Max) | 25.4 (17.7, 53.0) | |
| Medical history | None | 18 | (69.2%) |
|  | Yes | 8 | (30.8%) |
| Comorbidity | None | 5 | (19.2%) |
|  | Yes | 21 | (80.8%) |
| Smoking history | None | 9 | (34.6%) |
|  | Yes | 17 | (65.4%) |
| SpO_2_ (on oxygen) | Mean±SD | 92.3±2.3 | |
| (%) | Median (Min, Max) | 94 (88, 94) | |
| SpO_2_ (on room air) | Mean±SD | 92.9±2.2 | |
| (%) | Median (Min, Max) | 93 (89, 96) | |
| Symptom: Dyspnea | Grade 0 | 5 | (19.2%) |
| (mMRC) | Grade 1 | 9 | (34.6%) |
|  | Grade 2 | 3 | (11.5%) |
|  | Grade 3 | 6 | (23.1%) |
|  | Grade 4 | 3 | (11.5%) |
| Symptom: Cough | None | 7 | (26.9%) |
|  | Yes | 19 | (73.1%) |
| Symptom: Sputum | None | 20 | (76.9%) |
|  | Yes | 6 | (23.1%) |
| Chest X-ray findings | No abnormality | 0 | (0.0%) |
|  | With abnormality | 26 | (100.0%) |
| Chest CT findings | Mean±SD | 9.0±3.9 | |
| (Total score) | Median (Min, Max) | 9 (1, 22) | |

BMI: body mass index, mMRC: modified Medical Research Council dyspnea scale

**Supplementary Table 3. Adverse events (AEs)**

|  | Open-label study | Randomized study | |
| --- | --- | --- | --- |
|  | TM5614  (n=26) | TM5614  (n=39) | Placebo  (n=36) |
| Total AE cases | 42 | 38 | 42 |
| Patients with at least one AE (%) | 19 (73.1) | 22 (56.4) | 23 (63.9) |
| AE cases in which a relationship with the investigational drug could not be ruled out | 17 | 11 | 13 |
| Patients | 10 (38.5) | 7 (17.9) | 10 (27.8) |
| Bleeding, nasal | 3 (11.5) | 0 (0) | 0 (0) |
| Bleeding, hemoptysis | 3 (11.5) | 0 (0) | 0 (0) |
| Bleeding, subcutaneous | 1 (3.8) | 1 (2.6) | 0 (0) |
| Bleeding, urine occult blood | 0 (0) | 0 (0) | 1 (2.8) |
| Bleeding, severe menstrual | 0 (0) | 0 (0) | 1 (2.8) |
| Constipation | 0 (0) | 2 (5.1) | 3 (8.3) |
| Diarrhea | 0 (0) | 0 (0) | 1 (2.8) |
| Abnormal liver function, ALT | 2 (7.7) | 1 (2.6) | 3 (8.3) |
| Abnormal liver function, AST | 2 (7.7) | 0 (0) | 0 (0) |
| Abnormal liver function, γ-GTP | 0 (0) | 0 (0) | 1 (2.8) |
| Abnormal liver function, others | 1 (3.8) | 2 (5.1) | 0 (0) |
| Arrhythmia | 0 (0) | 0 (0) | 1 (2.8) |
| Bradycardia | 0 (0) | 0 (0) | 1 (2.8) |
| Pneumonitis | 1 (3.8) | 0 (0) | 0 (0) |
| Skin eruption/eczema | 2 (7.7) | 2 (5.1) | 0 (0) |
| Genital herpes simplex infection | 0 (0) | 1 (2.6) | 0 (0) |
| Acute otitis media | 0 (0) | 0 (0) | 1 (2.8) |
| Elevation of D-dimer | 1 (3.8) | 2 (5.1) | 0 (0) |
| Hyperpotassemia | 1 (3.8) | 0 (0) | 0 (0) |
| AE cases in which the relationship is considered probable | 0 | 0 | 1 |
| Patients | 0 (0) | 0 (0) | 1 (2.8) |
| Bleeding, nasal | 0 (0) | 0 (0) | 1 (2.8) |

AE: adverse event, AST: aspartate aminotransferase, ALT: alanine aminotransferase, γ-GTP: γ-glutamyltranspeptidase

**Supplementary Figure 1. Total CT score of the semiquantitative scoring system for lung lesions (open-label study)**


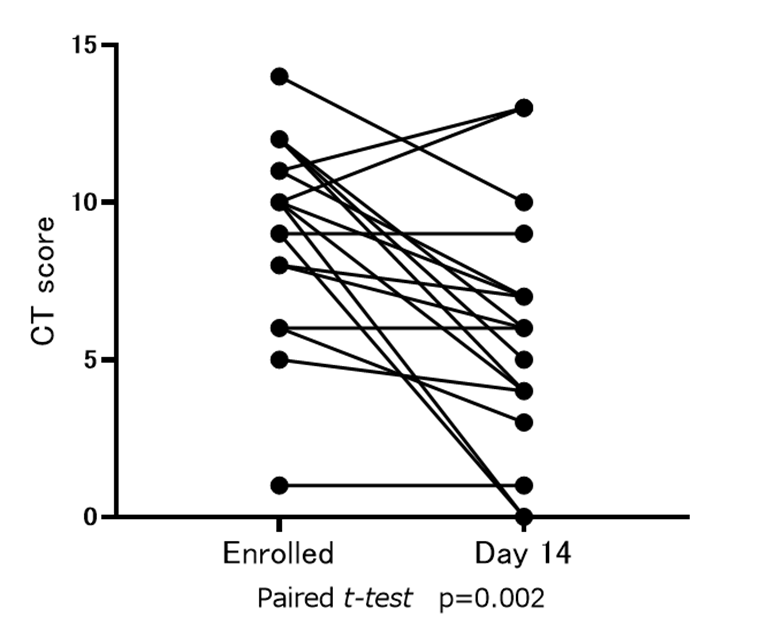


**Supplementary Figure 2. Normal lung area (%) on quantitative analysis of chest CT (randomized, double-blind, placebo-controlled study)**
